# Double blind randomized clinical trial comparing minimally- invasive envelope flap and conventional envelope flap on impacted lower third molar surgery

**DOI:** 10.4317/medoral.25425

**Published:** 2022-09-29

**Authors:** Samuel Macedo Costa, Bruna Campos Ribeiro, Alice Soares Gonçalves, Laura Maria de Almeida Araújo, Guilherme Lacerda de Toledo, Marcio Bruno Figueiredo Amaral

**Affiliations:** 1 DDS, OMFS, FBCOMFS, Ph.D. Researcher. Oral and Maxillofacial Surgery Phd Program, University of São Paulo FORP-USP, Ribeirão Preto, SP, Brazil; 2 Mater Dei Hospital Oral and Maxillofacial Surgery Service, Belo Horizonte, MG, Brazil; 3 DDS, OMFS Resident. Oral and Maxillofacial Surgery Residency Program, University of São Paulo FORP-USP, Ribeirão Preto, SP, Brazil; 4 DDS. College of Dentistry of the University Center of Belo Horizonte, UNIBH, Belo Horizonte, MG, Brazil; 5 DDS. Pontifical Catholic University of Minas Gerais, PUC-MG, Belo Horizonte, MG, Brazil; 6 DDS, OMFS. Head of the Mater Dei Hospital Oral and Maxillofacial Surgery Service, Belo Horizonte, MG, Brazil; 7 DDS, OFMS, Ph.D. Head of the Oral and Maxillofacial Surgery Residency Program, Hospital João XXIII/FHEMIG, Belo Horizonte, MG, Brazil

## Abstract

**Background:**

The latest trend in surgery is to look for minimally invasive procedures, with fewer complications and a shorter recovery time. This study aims to compare the minimally- invasive envelope flap, with smaller incision and fewer dissection and the conventional envelope flap, with a 20mm incision, on impacted mandibular third molar surgery, focusing on the hypothesis that there were no differences in postoperative outcomes.

**Material and Methods:**

A double-blind randomized clinical trial was designed to compare both incisions, focused on determining the approach with minor postoperative side-effects and minor impact on quality-of-life. A total of 60 patients were enrolled for the study if their presented impacted mandibular third molar and was 18-years-old or more. Both groups were evaluated from time elapsed on the surgery, maximum mouth opening, swelling and quality of life assessment.

**Results:**

The flap choice influenced facial swelling (*p*=0,03), pain on the first three days (*p*=0,037), interference with oral hygiene (*p*=0,019) and discomfort on speech (*p*=0,07). Chewing, swallowing, trismus, pain after seven days, postoperative complications and other quality-of-life arrangements were no different between groups.

**Conclusions:**

The minimally- invasive envelope flap could lead to a less painful experience for the patient, with fewer impact on the oral hygiene and speech discomfort.

** Key words:**Third molar, oral surgery, surgical procedure.

## Introduction

Impacted third molars are commonly observed in the oral and maxillofacial surgeon’s practice and their extraction is one of the most frequent surgical procedures in routine oral surgery (1). The indications for the surgical removal of the third molar are many, including pericoronitis (2), caries (3), infection (4), periodontitis (4,6-8), cysts or tumors (9-12), lack of function (5), prosthesis (5), orthodontics (13) and ortho-surgical treatment (5). The main causes for impaction are related to lack of space, therefore, usually, the majority of these should be removed (14).

The surgeon’s technique is one of the factors that influence postoperative morbidity and the patient's perception, together with that the level of tooth impaction, elapsed surgical time, age and even the presence of comorbidities could also have their importance (14).

The surgical flap should provide visibility and optimal access and its design and extension are directly related to post-operative pain, being the conventional envelope flap considered superior to many other proposed priory (15,16), however, this is still a place for controversy (16,17). The latest trend in surgery, all fields respected, including oral and maxillofacial surgery is to look for minimally invasive procedures, with fewer complications and a shorter recovery time.

A promising and easily replicable technique has been described by Khiabani and colleagues (15) for the less-invasive management of the impacted mandibular third molar, presenting excellent results on postoperative pain, swelling, mouth opening limitation, and therefore the quality of life, however, their study had a split-mouth design, not a broad randomized clinical trial.

This study aims to compare the minimally- invasive envelope flap and the conventional envelope flap on impacted mandibular third molar surgery, focusing on the hypothesis that there were no differences in postoperative outcomes. The group focused on determining if the proposed approach: 1) Reduce postoperative side effects, mainly pain, swelling, and mouth opening limitation. 2) improve the quality of life in the post-operative time. 3) Could be considered a faster realization technique.

## Material and Methods

To achieve the purpose of this study, the investigators designed a prospective double-blind randomized clinical trial. All the patients presenting to the group’s private practice with the indication for the removal of the mandibular third molar from September 2021 to January 2022 were considered eligible to receive the invitation to participate in the study. The study was submitted and approved by the Ethical Committee, under the IRB approval number 92432218.2.0000.5119, and registered on the Brazilian Clinical Trials Registry 202111170 (REBEC). The study was conducted in accordance with the declaration of Helsinki and followed all the protocols of the Consolidated Standards of Reporting Trials (CONSORT) Statement. All the patients received information regarding the study before signing the consent forms.

- Patient Selection

Patients were included in the study if 1) The patient agreed to participate; 2) 18 years old or more; 3) Presented impacted mandibular third molar, groups A, B, or C, and class 1 or 2 impaction according to Pell and Gregory classification; 4) Patients that agreed to remove their molars under local anesthesia in a single section.

Patients were excluded as subjects of the study if 1) The patient did not agree to participate; 2) Presence of any systemic disease; 3) Regular use of medication, except contraceptive drugs for women; 4) Abnormal impaction, requiring adjuvant procedures; 5) Presence of infection; 6) Presence of pericoronitis; 7) Presence of cysts or tumors; 8) Allergy to any of the drugs prescribed on the protocol; 9) Patient do not follow the protocol.

- Study Design

The study was proposed with a two-group design, Group A for the conventional envelope flap (CEF) and Group B for the minimally invasive envelope (MIE). All the surgical procedures were performed by the same surgeon (S.M.C.) with an assessor that was not related to this study. The only difference between both groups was the flap design.

All the patients were priorly evaluated by two independent analysts (G.L.T. and M.B.F.A.), who determined the patient inclusion criteria and allocated the patient’s mandibular third molar in the Pell and Gregory’s classification.

The enrolled patients were equally randomized between both groups using an automated non-influencing tool (Research Randomizer Version 4.0, Lancaster, USA) and only the surgeon (S.M.C.) had contact with the patient group prior to and after the surgery. The patient and the evaluators (A.S.G. and L.M.A.A.) had no contact with the list, or the patient surgical site prior, during, or after the procedure. The statistical expert (B.C.R.) also had no contact with the patient’s data, such as names, allocation data, or even outcomes.

The sample size was calculated on a normal mathematical operation using n=N.Z2.p.(1-p) / Z2.p.(1-p) + e2.N-1, being N= sample, Z= variable, *p*= event probability and e= error. In a 5% error tolerance, with 95% confidence interval, *p*=0,05.

- Surgical Technique

Group A- Conventional Envelope Flap

**Figure 1:**
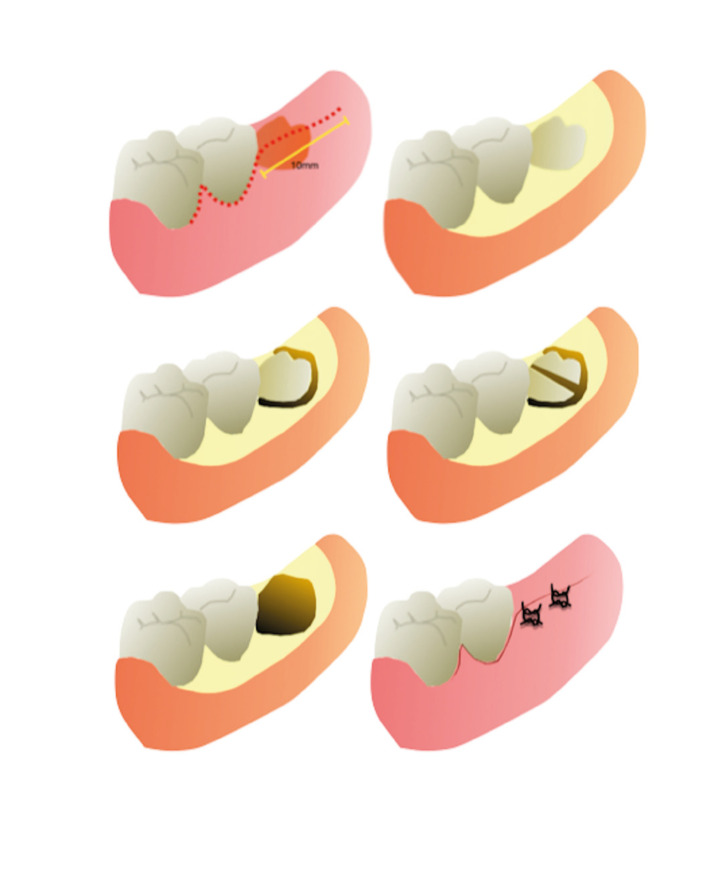
Schematic representation of conventional envelope flap, from incision to sutures.

Patients received 5mL of 0,12% Chlorhexidine (Periogard, Colgate-Palmolive, New York, USA) for a one-minute rinsing before the surgical procedure. All the procedures were performed under local anesthesia using 2 cartridges of 1.8mL 4% Lidocaine 1:100.000 epinephrine. (Xylestesin, DFL, Rio de Janeiro, Brazil). A Minnesota retractor was used to promote visualization and the incision was carried from the anterior second molar papillae to the lateral posterior aspect of the second molar, followed by a 20mm incision (Fig. 1). The mucoperiosteal flap was retracted, followed by the bone removal and tooth sectioning with a straight hand-piece and under copious irrigation with sterile normal saline solution, for all patients. After the tooth removal and irrigation, the flap was repositioned and closed with single 4-0 nylon sutures (Shalon, São Luiz de Montes Belos, Brazil). The elapsed time was recorded in minutes from the incision to the end of the sutures (Fig. 1).

Group B- Minimally-Invasive Envelope Flap

**Figure 2:**
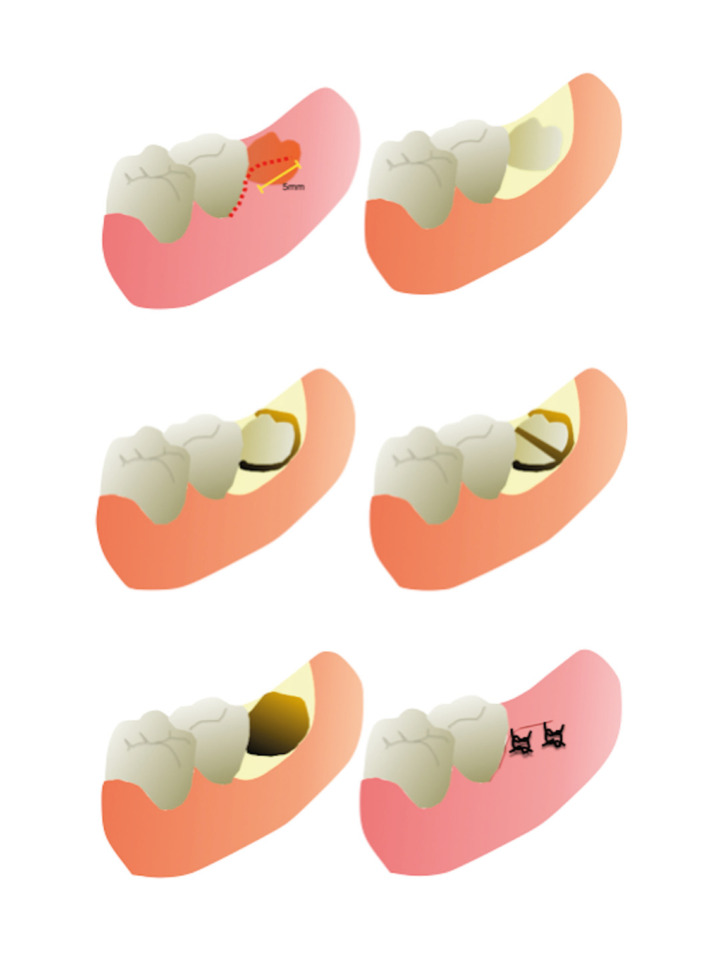
Schematic representation of minimally-invasive envelope flap, from incision to sutures.

The rinsing and anesthesia were carried out in the same protocol described for Group A. The incision in Group B was positioned from the medial aspect of the second molar gingival insertion to the posterior aspect of the second molar, followed by a 5mm incision (Fig. 2). The mucoperiosteal flap was retracted with caution, and the posterior aspect of the third molar was visualized under tissue tunnelization. The bone removal and sectioning with a straight handpiece and under copious irrigation with the sterile normal saline solution were performed also under tissue tunnelization for all patients. The tooth fragments were removed and after irrigation, the flap was repositioned and closed with single 4-0 nylon sutures (Shalon, São Luiz de Montes Belos, Brazil). The elapsed time was recorded in minutes from the incision to the end of the sutures (Fig. 2).

- Pharmacological Protocol

All the patients in both groups received the same drug protocol, with standard instructions. Amoxicillin (1g, 1-hour pre-op) and Dexamethasone (8mg, 1-hour pre-op), followed by Dexamethasone (4mg/8 h for 3 days) and Acetaminophen (500mg/8h for 5 days) on the postoperative period. Chlorhexidine mouthwash (4x day for 4 days) was also recommended for hygiene help.

- Clinical Assessment and Outcomes

The variables collected for the study were sex, age, the time elapsed on the surgery, maximum mouth opening, swelling, and quality of life assessment. The primary outcome variable was postoperative pain, recorded in two moments, T1: third postoperative day and T2: seventh postoperative day, using the subjective measurement of the Visual Analogue Scale (VAS). The pain was referred by the patient between 0 (no pain) to 10 (worst pain experienced).

The secondary outcome variables were postoperative side effects, including swelling and mouth opening limitation. Two independent and blinded measurers conducted the clinical measurements of maximum mouth opening and facial swelling on T0: preoperative time and T2: seventh postoperative day. Facial swelling was recorded using a tape measuring method through 3 facial lines while the patient was sitting straight at a 90˚ angle, and the mandible was at rest. These lines include the tragus distance to the corner of the mouth as line A, the tragus distance to soft tissue pogonion as line B, and the distance of the outer corner of the eye to the angle of the mandible as line C. The facial measurement was calculated as (A + B + C)/3, and facial swelling (%) was calculated as: ([postoperative measurement on T2 − preoperative measurement]/preoperative measurement) X 100 (15). The maximum mouth opening was recorded, measuring the maximum distance between the maxillary incisor's incisal edge to the lower incisor (to the nearest mm) using a metal ruler. Mouth opening limitation (MOL) was calculated as (preoperative measurement − postoperative measurement on T2 /preoperative measurement) X 100 (15).

Complications related to the surgery, flap laceration, wound dehiscence, infection, bleeding, alveolitis or nerve injury, intraoperative or postoperative, were recorded in both groups.

All the patients received a questionnaire in T1 and T2 to assess their postoperative quality of life. The questionnaire for the patients' postoperative quality-of-life scores (PPOQL) was used, to express the discomfort of the postoperative time and how the surgery affected their quality of life (15). The questionnaire was divided into five questions, including speech, dissatisfaction with appearance, pain perception, feeling of sickness, interference with oral hygiene, and activities, which according to the VAS, each scored from 0 to 10. Chewing and Swallowing impairment were also collected from the patient's perception in T1 and T2, in a 0 to 10 score.

- Data Analysis

All the data was recorded and tabulated and presented to the statistics expert (B.C.R.), without any means to identify the patients, allocation, and outcomes. As for descriptive analysis, appropriate charts and Tables were used to display the central tendency and dispersion indexes. The data distribution was evaluated with the Kolmogorov-Smirnov Test (KST). The normal distribution of the data provided the paired-sample t-test in order to compare the differences between both groups. The significance level was set at 0.05 using SPSS 23 (SPSS Inc, IBM, Armonk, NY).

## Results

- Demographics

A total of 71 subjects were enrolled for this study and sixty agreed to participate in the study or meet the inclusion criteria. The parallel design of the study subdivided thirty subjects in each group. An equal number of male and female patients were observed in each group.

The mean age among the subjects was 21,63 (± 5,14) years, from 18 to 40 years old. In both groups the mean age was similar, 21,65 (± 4,76) in Group A and 21,73 (± 5,57) in Group B.

The duration of the surgical procedure was calculated as described in the methods and the mean time was 11,69 (± 7,69) minutes. For Group A was measured 14,00 (± 8,69) minutes and 9,37 (± 5,83) minutes for Group B, in which the statistical difference reached significance (*p*= 0,048).

The demographic information and data are presented in Table 1.

- Pain, Chewing, and Swallowing

The results for both pains, chewing, and swallowing was recorded on the third and seventh postoperative days. For both groups, the pain perceptions decreased on the VAS from the 3rd day to the 7th day (*p*=0,003). However, the pain perception was significantly reduced in the minimally invasive group during the first three days (*p*=0,037). The postoperative pain level on the seventh day was (1,86, ± 2,07) in the Group A and (2,41, ± 2,63) in the Group B (*p*=0,059).

The difficulty to chew during the postoperative time was measured by the patient and on the Group A was (6,29,± 2,37), followed by (4,26, ± 2,47) in the Group B on T1. On T2, Group A (3,34, ± 2,72), followed by 2,06,± 2,65 in Group B. For swallowing, both groups presented similar values on the post-operative time on T2, (0,51, ± 1,51), (0,43, ± 1,14) respectively. Those results were not significant for both groups' comparison (Table 2).

**Table 1:**
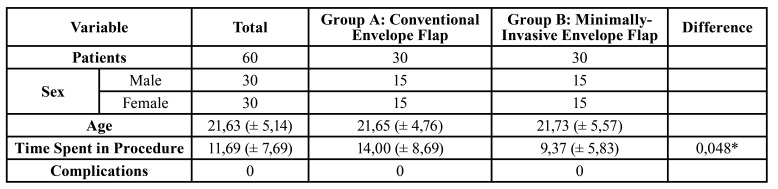
Descriptive analysis between groups.

- Facial Swelling and Mouth Opening Limitation

The data for facial swelling and mouth opening limitation is presented in Table 2, together with data from pain, chewing, and swallowing.

The groups presented an expressive difference between groups on swelling on T2, Group A presented a value of 5,84 %, ± 4,68. On the other hand, Group B presented a significantly decreased value of 2,64%, ± 4,75 (*p*=0,03).

Both Groups presented a similar impact on the mouth opening, being Group A less impaired (11,68%, ± 13,68), followed by 16,48%, ± 12,5 in Group B. However, the flap design does not meet the statistical significance (*p*= 0,799).

- Perception of Quality of life [PPQL]

The quality of life was assessed by ten sub-scales, and Table 3 describes the PPQL data. The results revealed differences between the groups in only two subscales, Discomfort in Speech on the 7th postoperative day and Interference on Hygiene on the third-day post-surgery. Focusing on PPQL, both flaps are equal on the patient perception, both on the T1 and T2 (Table 3).

**Table 2:**
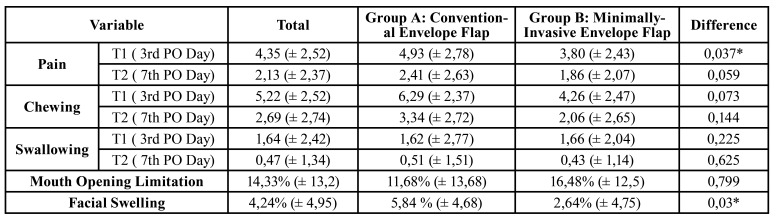
Pain, chewing, swallowing, mouth opening limitation and facial swelling.

**Table 3:**
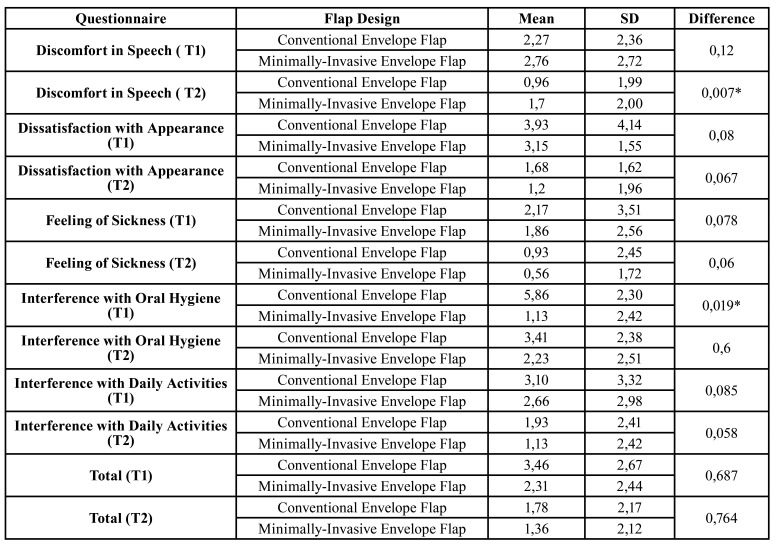
Perception on quality of life (ppql).

- Complications

Regardless of the group, no cases of transoperative or postoperative complications were observed among the study patients.

## Discussion

The aim of this study was to compare a traditional approach for the exposure of the lower third molar and a modified one, the minimally- invasive envelope flap, focusing on postoperative discomfort, mainly pain, swelling, and mouth opening limitation. The impact of the procedure on the patient's perception of quality of life and the mean time of surgery was also evaluated and compared.

This trial had some natural limitations, as the patients are from the same city and had equally comparable conditions to achieve postoperative care and instructions, for more heterogeneous populations, the results could not be replicable. The group focused on avoiding bias in the allocation and blinding of the patients, which could contaminate the results and the analysis. For both the evaluators, none was able to determine the patient allocation during or even after the study.

A series of multiple types of incisions and flap designs have been described in the literature, aiming for a more comforTable postoperative period for the patients, with a reduction in pain, swelling, and trismus (17-19). The ideal flap should provide full visibility, and accessibility, with minor impact on adjacent structures. Other variables play an important role in the postoperative outcome after third molar surgery, such as degree and type of impaction, amount of bone to be removed, and the time spent for the procedure, is the last one reported as the most important (20).

The conventional envelope flap was first described by Szmyd in 1971 (20) and is by far the most commonly used for impacted third molar surgeries and is considered easier to perform, reducing postoperative surgical complications (17). The minimally-invasive envelope flap, described by Khiabani and colleagues (15), focused on the philosophy of small incisions and less dissection, for a complication-free shortest recovery time possible. In their initial study, the MIE promoted a noticeable improvement in postoperative consequences, reducing pain, swelling, trismus, and impact on the PPQL (15).

This double-blind randomized clinical trial compared the traditional conventional envelope flap and the minimally-invasive envelope flap on the variables related to the postoperative period, PPQL, and time spent in surgery. The mean age and sex distribution were similar in both groups. The MIE showed to be a simple technique, feasible, and with a minor surgical time (*p*=0,048), which was considered impressive, due to the smaller exposure. The longer the procedure, the more biological pro-inflammatory mediators will be released, influencing the patient postoperative outcome (20).

On the third postoperative day, the impact of the MIE was expressive for pain perception on the VAS scale, probably due to the inflammatory phase of the recovery and cicatrization periods. On T2, the seventh postoperative day, both groups were no differences in pain perception, chewing, and swallowing impairments. Mouth opening limitation is considered one of the most uncomforTable points in the postoperative period of oral surgery, especially in an impacted lower third molar. Both groups presented similar results on the mouth opening impairment, corroborating findings that other variables play a fundamental role in the MOL, such as bone removal and dental section (17,20). In this study, all the patients presented impacted third molars, that required both bone removal and dental section with a straight hand-piece.

Facial swelling is a difficult variable to measure, and bias could be present even with calibrated examiners (17). This study used the same tool performed in the Khiabani article. The MIE was superior to the conventional envelope flap, promoting less edema (*p*=0,03) and confirming that the surgical time is fundamental in the installation of the inflammatory condition, with more swelling (17,20). Along with this, small incisions, with fewer dissection and detachment are expected to have a better prognosis for swelling, due to the avoidance of a relief incision (17). Incision designs with larger flap release, especially beyond the buccinator attachment, results in more swelling in the space of the body of the mandible, and that extension of the flap into the temporalis tendon results in more trismus.

The patient's perception of quality of life is the final stance of the perception of a good and comforTable postoperative period. Both incisions are equal in promoting a healthy recovery with no direct impact on quality of life. On the other hand, the patients had interference with oral hygiene in the first three days, due to the obvious difficulty caused by the procedure and initial swelling. After seven days, the patients presented discomfort with speech in the group that received the MIE, however, the mean values for both groups are considered insignificant (1,7 on 10, VAS).

For both groups, no complications were observed in the study, such as infection or alveolar osteitis. Other studies compared flaps with the conventional envelope (20) focusing on postoperative complications and no difference was observed.

Many studies focused on oral surgery and especially on mandibular third molar management. Systematic reviews of the meta-analysis suggested that the flap itself does not influence pain, swelling, trismus, and postoperative complications like dehiscence or alveolar osteitis (17). The CEF presented better results for the exposure of the impacted third molar than other flaps, in particular the triangular flap (20). Khiabani’s minimally-invasive flap followed the philosophy of smaller incisions and less tissue aggression, being considered promising (15), however, in that study, the mean time for the surgery was more than double than observed for the MEF in this study (20 min vs 9,37 min).

In conclusion, this study revealed that the minimally-invasive envelope approach is reliable and feasible, and easy to perform, however, the inexperienced professional may find it difficult to retract and remove the tooth under strict tissue tunnelization. The technique is faster than the CEF in the meantime, which promoted nice results on the postoperative pain in the first three days and facial swelling. The conventional envelope flap remains the gold standard for the exposure and removal of the impacted lower third molar, however, with more studies comparing both incisions the minimally-invasive flap could arise as an optimal option.

## References

[1] Bonetti GA, Bendandi M, Laino L, Checchi V, Checchi L (2007). Orthodontic extraction: riskless extraction of impacted lower third molars close to the mandibular canal. J Oral Maxillofac Surg.

[2] Rakprasitikul S (2001). Pathologic changes in the pericoronal tissues of unerupted third molars. Quintessence Int.

[3] Nordenram A, Hultin M, Kjellman O, Ramstrom G (1987). Indications for surgical removal of the mandibular third molar: study of 2,630 cases. Swed Dent J.

[4] Dodson TB (2012). Surveillance as a management strategy for retained third molars: is it desirable?. J Oral Maxillofac Surg.

[5] Steed MB (2014). The indications for third-molar extractions. The Journal of the American Dental Association.

[6] Blakey GH, Marciani RD, Haug RH, Phillips C, Offenbacher S, Pabla T (2002). Periodontal pathology associated with asymptomatic third molars. J Oral Maxillofac Surg.

[7] White RP Jr, Offenbacher S, Phillips C, Haug RH, Blakey GH, Marciani RD (2002). Inflammatory mediators and periodontitis in patients with asymptomatic third molars. J Oral Maxillofac Surg.

[8] White RP Jr, Madianos PN, Offenbacher S, Phillips C, Blakey GH, Haug RH (2002). Microbial complexes detected in the second/third molar region in patients with asymptomatic third molars. J Oral Maxillofac Surg.

[9] Kinard BE, Dodson TB (2010). Most patients with asymptomatic, disease free third molars elect extraction over retention as their preferred treatment. J Oral Maxillofac Surg.

[10] Güven O, Keskin A, Akal UK (2000). The incidence of cysts and tumors around impacted third molars. Int J Oral Maxillofac Surg.

[11] Song F, O'Meara S, Wilson P, Golder S, Kleijnen J (2000). The effectiveness and cost-effectiveness of prophylactic removal of wisdom teeth. Health Technol Assess.

[12] Hicks EP (1999). Third molar management: a case against routine removal in adolescent and young adult orthodontic patients. J Oral Maxillofac Surg.

[13] Marciani RD (2007). Third molar removal: an overview of indications, imaging, evaluation, and assessment of risk. Oral Maxillofac Surg Clin North Am.

[14] Chu H, Li Z, Ren F, Yang Z, Wu Z, Rong M (2020). Clinical application of flap or flapless buccal surgery on the extractions of mesially/horizontally impacted 3rd molar with high or medium position impact: A comparative study. J Stomatol Oral Maxillofac Surg.

[15] Khiabani K, Amirzade-Iranaq MH, Babadi A (2021). Does Minimal-Invasive Envelope Flap Reduce Side Effects Compared to Conventional Envelope Flap Following Impacted Third Molar Surgery? A Split-Mouth Randomized Clinical Trial. J Oral Maxillofac Surg.

[16] Glera-Suárez P, Soto-Peñaloza D, Peñarrocha-Oltra D, Peñarrocha-Diago M (2020). Patient morbidity after impacted third molar extraction with different flap designs. A systematic review and meta-analysis. Med Oral Patol Oral Cir Bucal.

[17] Da Silva BCL, Machado GF, Primo Miranda EF, Galvão EL, Falci SGM (2020). Envelope or triangular flap for surgical removal of third molars? A systematic review and meta-analysis. Int J Oral Maxillofac Surg.

[18] Isola G, Matarese M, Ramaglia L, Cicciu M, Matarese G (2019). Evaluation of the efficacy of celecoxib and ibuprofen on postoperative pain, swelling, and mouth opening after surgical removal of impacted third molars: a randomized, controlled clinical trial. Int J Oral Maxillofac Surg.

[19] Zhu J, Yuan X, Yan L, Li T, Guang M, Zhang Y (2020). Comparison of Postoperative Outcomes Between Envelope and Triangular Flaps After Mandibular Third Molar Surgery: A Systematic Review and Meta-Analysis. J Oral Maxillofac Surg.

[20] Szmyd L (1971). Impacted Teeth. Dent Clin North Am.

